# Erratum to: Diet Modulates the Effects of Genetic Variants on the Vitamin D Metabolic Pathway and Bone Mineral Density in Mexican Postmenopausal Women; doi: 10.1093/jn/nxab067

**DOI:** 10.1093/jn/nxab166

**Published:** 2021-06-01

Address correspondence to RV (e-mail: rvelazquez@inmegen.gob.mx).

Erratum to: Diet Modulates the Effects of Genetic Variants on the Vitamin D Metabolic Pathway and Bone Mineral Density in Mexican Postmenopausal Women; doi: 10.1093/jn/nxab067.

In the originally published version of this manuscript, the affiliation of the corresponding author was incorrect. Dr Rafael Velázquez-Cruz's correct affiliation is: Genomics of Bone Metabolism Laboratory, National Institute of Genomic Medicine (INMEGEN), Mexico City, Mexico. This error has been corrected.

